# Patient perspective on the elimination mother-to-child transmission of HIV, syphilis, and hepatitis B in Bali, Indonesia: a qualitative study

**DOI:** 10.1186/s12889-024-19692-3

**Published:** 2024-08-20

**Authors:** Luh Nik Armini, Elsa Pudji Setiawati, Nita Arisanti, Dany Hilmanto

**Affiliations:** 1 Doctoral Study Program, Faculty of Medicine, Universitas Padjadjaran, Sumedang, 45363 Indonesia; 2 Midwifery Science Program, Faculty of Medicine, Universitas Pendidikan Ganesha, Bali, 81116 Indonesia; 3 Department of Public Health, Faculty of Medicine, Universitas Padjadjaran, Sumedang, West Java 45363 Indonesia; 4 Department of Child Health, Faculty of Medicine, Universitas Padjadjaran, Sumedang, 45363 Indonesia

**Keywords:** EMTCT, HIV, Hepatitis B, Syphilis, Facilitators, Barriers

## Abstract

**Background:**

This study aimed to explore the facilitators and barriers to the elimination of human immunodeficiency virus (HIV), syphilis, and hepatitis B transmission based on the perspectives of mothers living with HIV, syphilis, and hepatitis B.

**Methods:**

This study employed a descriptive, qualitative design. Semi-structured interviews were conducted with mothers living with HIV, syphilis, and/or hepatitis B virus. A total of 25 participants were included in the study. This study used a triangulation method conducted by members to enhance the validity and dependability of the findings. The study was conducted at referral hospitals and community health centers between September 2022 and February 2023. Data analysis utilized deductive content analysis and categorized themes based on a socio-ecological framework.

**Results:**

The findings revealed facilitators and barriers across five levels of the socio-ecological framework and 21 subcategories. The findings included the following: (1) At the policy level, facilitators were mandatory testing programs, and barriers were separating testing services from antenatal care facilities. (2) At the community level, facilitators included the involvement of non-governmental organizations (NGOs) and cross-sector support. Barriers included challenges faced by non-residents and fear of stigma and discrimination. (3) At the healthcare system level, facilitators included tracking and follow-up by midwives, positive relationships with healthcare providers, and satisfaction with healthcare services. Barriers included prolonged waiting times, insufficient information from healthcare providers, and administrative limitations. (4) At the interpersonal level, facilitators included partner and family support, open communication, and absence of stigma. Barriers included the reluctance of sexual partners to undergo screening. (5) At the individual level, facilitators included the desire for a healthy baby, adequate knowledge, self-acceptance, and commitment to a healthy lifestyle; barriers included the lack of administrative discipline.

**Conclusion:**

Mothers living with HIV, syphilis, or hepatitis B require tailored healthcare approaches. Healthcare professionals must understand and meet the needs of mothers within a comprehensive care continuum. The findings of this study advocate for the development and implementation of integrated care models that are responsive to the specific challenges and preferences of affected mothers, aiming to improve health outcomes for both mothers and their children.

**Supplementary Information:**

The online version contains supplementary material available at 10.1186/s12889-024-19692-3.

## Background

The triple elimination program for mother-to-child transmission (EMTCT) of human immunodeficiency virus (HIV), syphilis, and hepatitis B in Indonesia began in 2018 through a policy issued by the Ministry of Health of the Republic of Indonesia in 2017. EMTCT programs are integrated into antenatal care (ANC) [1]. Although hepatitis B may not be sexually acquired, these three diseases have the same mode of transmission from mother to child. Hence, efforts toward elimination can be carried out together to provide benefits and efficiency in its implementation [2, 3]. Although policies and strategies to achieve triple elimination have been established and implemented, pregnant women living with HIV, syphilis, and/or hepatitis B face challenges in preventing transmission of infection from mother to child; many pregnant women are screened late, even before delivery, and do not receive treatment [4, 5]. In Bali Province, EMTCT achievements in three districts have not achieved the target set by the World Health Organization (WHO) and the Ministry of Health and have faced challenges [6]. Treatment of pregnant women living with HIV, syphilis, and/or hepatitis B was far below the target despite screening of pregnant women who exceeded WHO targets [7]. A study in Indonesia revealed that one-third of pregnant women living with HIV never initiated ART treatment, only half of those who initiated treatment remained on ART, and ART treatment retention among pregnant women was greater in tertiary and secondary hospitals [8].

The WHO advocates for EMTCT through a comprehensive approach spanning prevention, diagnosis, treatment, and care [9]. Health systems and programs need to ensure that all pregnant women living with HIV, syphilis, and hepatitis B, along with their infants, are effectively treated and that their sexual partners also undergo examination and treatment [10]. The set The WHO global target for EMTCT validation at 95% for all infections, 50 per 100,000 live births for HIV- and syphilis-infected children, and ≤ 2% for the hepatitis B MTCT rate. The national targets to end the transmission of HIV, syphilis, and hepatitis B have not been achieved by 2022. In Indonesia, only approximately 54% of the mothers are screened for HIV infection. The number of babies born to HIV-positive mothers was 10%, the number of children who received prophylaxis was 103 (76.87%), and the number of children who received ART was 26 (19.4%) [11]. Among 35,757 children born to mothers with hepatitis B infection in 2022, only 27% (9,239 children) were screened, and approximately 1.46% (135 children) were hepatitis B-positive. In 2016, there were approximately 661,000 children with congenital syphilis in 2016. The decline in congenital syphilis is slow, and there is a gap between hepatitis B screening and the management of HBsAg-positive patients [12]. Therefore, antenatal testing among pregnant women and Indonesia's impact target (case rate among children) is still below the expected global target.

The WHO also expects each country to tailor its actions to achieve EMTCT to local conditions while ensuring fair access and respecting human rights, with care centered on individuals, especially pregnant or postpartum mothers [4]. This study aimed to explore the facilitators of and barriers to the elimination of HIV, syphilis, and/or hepatitis B transmission from mother to child from the perspective of infected mothers. The findings of this study may help to characterize mothers and provide insights into improving EMTCT services. This study may also be utilized in providing women-centered care services, enabling precise interventions for infected mothers based on the challenges they face, and facilitating the success of preventing this infection from mother to child. Moreover, this study may provide insights into gender equality in efforts to eliminate mother-to-child transmission of HIV, syphilis, and hepatitis B by providing mothers with an enabling environment and social support. This highlights the need for a holistic approach to address ongoing gender inequalities. Public health programs should specifically target these issues in order to achieve effective elimination.

## Methods

### Study design

This study was designed as a descriptive qualitative study. [13] Semi-structured qualitative interviews were conducted with postpartum mothers living with HIV, syphilis, and/or hepatitis B to explore the facilitators of and barriers to the elimination of infection transmission from mother to child.

### Study setting

The study was conducted from September 2022 to February 2023 at two referral hospitals and five public health centers. This study was conducted over a period to capture a diverse range of experiences and responses from the participants, thereby enhancing the reliability and depth of the study findings.

### Sample size

A purposive sampling strategy was used to select the participants. The sample in this study comprised 25 participants, including mothers living with HIV, Syphilis, and Hepatitis B. This sample was reached according to saturation.

### Inclusion and exclusion criteria

This study included postpartum mothers who were newly diagnosed with HIV, syphilis, and hepatitis B and had experience in receiving EMTCT services, including screening, treatment during pregnancy, and prevention for the baby. Furthermore, the study exclusively included mothers who provided informed consent to participate. Newly diagnosed mothers were defined as those who were diagnosed at the recent stage of pregnancy and induced the EMTCT program. The accuracy of the testing history, maternal and infant treatment, and immunization status were cross-checked through the participants’ medical records and maternal and child health books. Additionally, mothers with severe mental health conditions that may affect their ability to participate were excluded.

### Recruitment participants

**Fig. 1:**
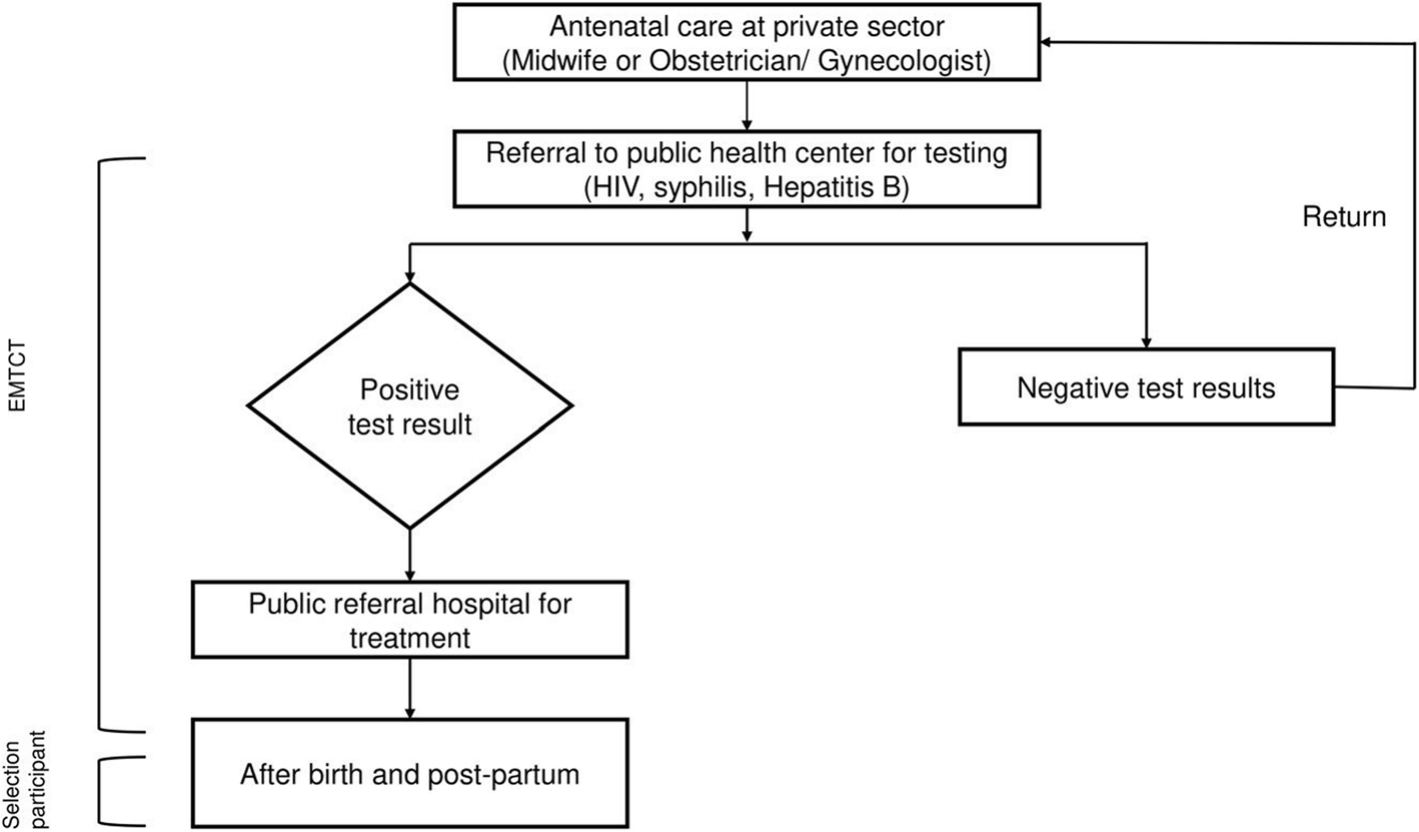
Rectruitment of partcipant’s diagram

The participants in this study included only mothers who met the inclusion criteria. Face-to-face interviews were conducted with participants at locations agreed upon by the interviewer. Participants were recruited until they reached a high level of saturation. Before receiving EMTCT services, primary maternal care can be provided in both private and public settings. However, patients who test positive are referred to public health services, whereas those who test negative continue antenatal care at their original primary care provider. Therefore, the sample selection was conducted only for public health services. The participant recruitment scheme is shown in Fig. 1.

### Data collection

This study was conducted after obtaining approval from the heads of the public referral hospitals and public health centers. Nineteen interviews were conducted in hospitals, and six were conducted at public health centers. The interviews were facilitated by the author (LNA) and an independent facilitator from a healthcare professional who had received interview and/or qualitative study training. This study was initiated by developing an interview guide designed to facilitate data collection using a series of semi-structured questions. These interview guidelines were developed through research (see Supplementary File 1). The development of the question guide was based on the social-ecological framework model and a review of the literature on factors influencing the elimination of the transmission of three infectious diseases, HIV, syphilis, and hepatitis B, from mother to child [14–16]. Separate interview guides were created for mothers living with HIV, syphilis, and hepatitis B, taking into consideration different contexts and experiences. Recognizing the potential for co-infections among participants, we used only one of them in the interview guide. The interview guide was designed for Indonesia. The interviewers were fluent in Indonesian and Balinese and had a qualitative interview experience. The interview sessions lasted approximately 30–60 min. The data collectors were midwives who assisted in identifying respondents who met the study criteria and had received comprehensive training. This training focused on the nuances of using the interview guide, ethical considerations, and techniques for capturing context and nuances beyond audio recordings such as supplementary notes. After ensuring that all data collectors were adequately prepared, interviews were conducted with the approval of the heads of the public referral hospitals and public health centers. The interviews were audio-recorded to ensure accuracy and supplementary notes were taken to capture any contextual details or nonverbal cues that were not evident in the audio recordings. Throughout this process, quality assurance measures were applied rigorously to maintain the integrity and reliability of the collected data.

### Data analysis

Descriptive statistical analysis was used to calculate the frequency and percentage of participants' sociodemographic data, which provided information on their characteristics. The study conducted deductive content analysis to identify emerging themes and used existing theories or literature to guide and identify variables or concepts of interest [17, 18]. Data saturation was reached with a total of 25 participants [15]. The interviews were transcribed verbatim, and the transcripts were coded using NVivo 12.0 software. The transcripts were coded line-by-line for data analysis, emphasizing the patient’s barriers and facilitator factors of continuity care toward triple EMTCT. The researchers independently coded and integrated the codes, when necessary. Potential main themes were derived by fusing similar codes into subthemes. Categories were grouped based on overarching themes and mapped onto [19]. The socioecological health model, which is also known as the socioecological model, a framework developed to understand the multifaceted influences on human behavior. This model considers factors at various levels, from the individual to the broader community and policy contexts [19]. The use of socioecological models in the context of infectious disease is well established. Public health studies have previously utilized this framework to explore barriers and facilitators for mothers living with HIV in care retention and HIV-exposed infant testing [20, 21]. This study identified five categories and 21 subcategories. This category refers to the socio-ecological framework. Facilitators and barriers that influence treatment adherence were identified in this subcategory.

### Trustworthiness

This reliability was evaluated using four criteria: credibility, dependability, transferability, and confirmability [22]. Two researchers established credibility by initially categorizing the data, which were reviewed and restructured by a third researcher. In addition, specific interview transcripts were returned to participants for clarification. At this stage, the author ensured that the participants and peer researchers had potentially stigmatizing narratives. Participants concurred with the content and interpretation of the transcripts. Continuous evaluation and assessment of the significance and completeness of data ensured dependability. Confirmability was attained through the categorization of coding into distinct groups, and all researchers collectively deliberated on the main groups. A single researcher with expertise in qualitative research and auditing oversaw the entire auditing process. Concurrently, deepening of the data was made possible through semi-structured interviews. A comprehensive exposition was provided regarding all stages of data acquisition and analysis as well as the participants to enhance transferability. Moreover, this study was conducted by members (PhD qualifications), and data checking using the triangulation method enhanced the validity and dependability of the findings. The researchers assessed their role in the data collection and analysis in a reflective manner. The research team engaged in discussions about the data, codes, and themes. It comprised of a midwife, pediatrician, family physician, and public health practitioner. The interviews were followed by debriefing sessions for the researchers.

## Results

### Characteristics of the participants

A total of 25 participants were interviewed, comprising mothers infected with HIV (30.33%), mothers infected with syphilis (33.33%), and mothers infected with hepatitis B (36.37%). Sociodemographic characteristics including age, education, occupation, parity, marital status, pre-existing conditions, partner infection status, and place of residence are presented in Table 1.

**Table 1:** Characteristic of participant

Variable	Frequency (n)	Percentage (%)
Age (year)		
< 20	2	8
20–35	20	80
> 35	3	12
Education level		
Grade 1–9	4	16
Grade 10–12	18	72
Collage	3	12
Occupation		
Housewife	11	44
Farmer	5	20
Civil servant	9	36
Parity		
Primipara	11	44
Multipara	14	56
Marital status		
Married, legally recognized	22	88
Married, not legally recognized	2	8
Cohabiting, not married	1	4
Disease		
HIV	6	24
Syphilis	8	32
Hepatitis B	9	36
HIV + Hepatitis B	2	8
Partner status		
Positive	8	32
Negative	6	24
Unknown	11	44

### Patient perspective on their care of triple EMTCT

**Fig. 2:**
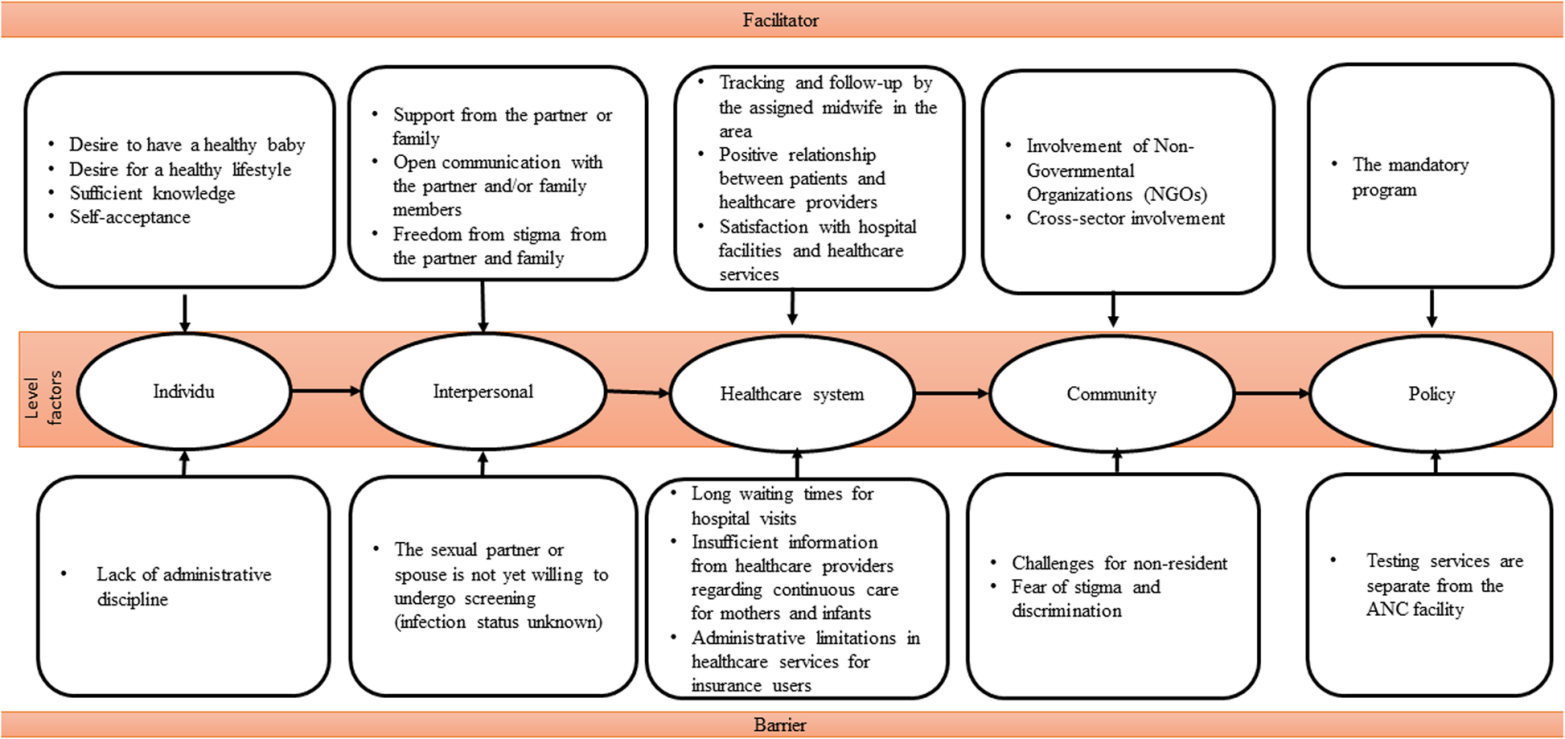
Barriers and Facilitators of EMTCT in Bali Province, Indonesia

This study revealed five categories following a socio-ecological framework: policy, community, healthcare system, intrapersonal, and individual. There were 21 subcategories encompassing facilitators and barriers. The categories and subcategories are presented in Fig. 2 and Table 2, respectively. Facilitator factors include (1) mandatory programs, (2) the involvement of nongovernmental organizations (NGOs), and (3) cross-sectoral involvement. (4) Tracking and follow-up by the assigned midwife in the area; (5) positive relationship between patients and healthcare providers; (6) satisfaction with hospital facilities and healthcare services; (7) support from the partner or family; (8) open communication with the partner and/or family members; (9) freedom from stigma from the partner and family; (10) desire to have a healthy baby; (11) sufficient knowledge; (12) self-acceptance; and (13) desire for a healthy lifestyle. Moreover, the barrier factors include (1) testing services separate from the ANC facility, (2) challenges for nonresidents, (3) fear of stigma and discrimination, (4) long waiting times for hospital visits, (5) insufficient information from healthcare providers regarding continuous care for mothers and infants, (6) administrative limitations in healthcare services for insurance users, (7) the sexual partner or spouse not yet willing to undergo screening, and (8) lack of administrative discipline.

**Table 2:** Category, sub-category, and quotations of study

Category	Sub-category	Quotations
Policy level factors	F: The mandatory program	Q1: "*I had my blood checked as advised by the midwife. She said pregnant women must have a complete blood test at the PHC so that the baby can be healthy and I can give birth at their facility*." (P3, Hepatitis B)
	B: Testing services are separate from the ANC facility	Q2: "*I have to go to the PHC for a comprehensive blood test. The place where I get tested does not offer the recommended blood tests*." (P14, Hepatitis B)
Community level factors	F: Involvement of Non-Governmental Organizations (NGOs)	Q3: "*I was assisted by the village head to obtain a certificate of inability to pay, so my KIS became active, and I received medical treatment, childbirth, and other services for free*." (P1, HIV)
	F: Cross-sector involvement	Q4: "*The companion provided a lot of informations, my parents and husband were also informed about HIV by that companion of mine*." (P11, HIV)
	B: Challenges for non-resident	Q5: "*I was checked at that PHC about two months ago, but I am still in the process of obtaining my ID card. They said to activate the KIS, I need to have a local ID card first*." (P24, Syphilis) Q6: "*I cannot obtain the KIS because I don't have a local ID card here. So when I seek treatment, I pay first, and afterward, I pay for private BPJS (insurance)."* (P12, HIV)
	B: Fear of stigma and discrimination	Q7: "*In the beginning, I was embarrassed and afraid to seek treatment, afraid that my neighbors would know about my illness*…" (P9, Syphilis)
Healthcare system or institutional-level factors	F: Tracking and follow-up by the assigned midwife in the area	Q8: "*A midwife visited my home to examine my Child Immunization Card (KIA) to determine if I had received treatment or not*" (P10, Syphilis) Q9: "*I was picked up by the midwife at my residence and taken to the PHC for a blood test*" (P7, Hepatitis B)
	F: Positive relationship between patients and healthcare providers	Q10: "*I underwent a blood test based on the advice of a doctor at the PHC, and I followed all the doctor's recommendations for treatment at the hospital*" (P21, Syphilis)
	F: Satisfaction with hospital facilities and healthcare services	Q11: "*Hospitals nowadays are different from the past. I was quickly attended to during childbirth, and now even the third-class rooms have air conditioning. Despite being far from home, it takes 1.5 h, I plan to give birth to my third child here. The doctors and medication are more comprehensive here*" (P23, Syphilis)
	B: Long waiting times for hospital visits	Q12: "*If you want to go to the hospital, it's a day out of my time..in the morning, you come to queue at the counter first, then at 10–11 o'clock you will be called to be asked to go to the clinic*" (P20 Hepatitis B)
	B: Insufficient information from healthcare providers regarding continuous care for mothers and infants	Q13: "*They only mentioned a blood test. I thought checking blood pressure was considered a blood test, so I didn't immediately go to the PHC*" (P8 HIV + Hep B) Q14: "*I wasn't informed that my baby needed further examination. The nurse only said that I needed to recheck the titer later*" (P24 Syphilis)
	B: Administrative limitations in healthcare services for insurance users	Q15: "*I cannot simultaneously pick up medication and have a prenatal checkup at the hospital in one day because I was told it's not allowed by BPJS. So, this week I'll go to the internal clinic, and two weeks later, I'll go to the obstetrics clinic*" (P25 Hep B)
Interpersonal level factors	F: Support from the partner or family	Q16: "*My husband works out of town, but he takes time off from work for every prenatal checkup and medication pickup. He consistently supports me in seeking medical care every month*" (P5, HIV)
	F: Open communication with the partner and/or family members	Q17: "*My father is more concerned than I am. When my medication is running out, he ensures that I have it, making sure I take the medicine if my husband can't accompany me. He is the one who takes me for checkups*" (P13, HIV)
	F: Freedom from stigma from the partner and family	Q18: "*I don't want to hide anything from my husband so that there's no burden on our hearts. Why lie about anything?*" (P4, Syphilis)
	B: The sexual partner or spouse is not yet willing to undergo screening (infection status unknown)	Q19: "*My husband is not willing to get tested; he's afraid of the potential results*" (P15, HIV)
Individual level factors	F: Desire to have a healthy baby	Q20: “*My main motivation is that I want my child to be born healthy and without any disabilities because I have experienced a miscarriage. So, whatever the doctor says, I always followed the medical advice and attended checkups*” (P9, Syphilis)
	F: The desire for a healthy lifestyle	Q21: "*I almost died when I didn't take my medication regularly, so now I want to be healthy and not experience sickness like before" (P13, HIV). Q22: "Before being injected with syphilis medication, I used to experience body aches to the bones. After receiving three injections, I feel healthy, and my body no longer aches*" (P4, Syphilis)
	F: Sufficient knowledge	Q22: “*I was informed that my illness could be transmitted to my child, so I was advised to seek treatment at the hospital”* (P16, Hep B) Q23: "*I don't know for how long my child will need to take medication; yesterday, I was just told that if we don't seek treatment, the child might get infected*" (P12, HIV)
	F: Self-acceptance	Q24: "*The doctor's examination revealed that I had a disease, so I followed the doctor's instructions to seek treatment at the hospital. Why delay treatment, so I went for it*" (P2, HIV + Hepatitis B)
	B: Lack of administrative discipline	Q25: "*I knew I was HIV positive for a while, but I delayed seeking medical attention until I obtained an ID card to register and KIS. So, it took about 1.5 months from when I learned about my positive status until I completed the ID card and obtained a letter of financial inability from the village. After that, it took an additional two weeks for the health insurance to become active*" (P8, HIV + Hep B)

*F* Facilitator, *B* Barrier

### Policy-level factor

Facilitators at the policy level are related to adherence to mandatory programs from healthcare professionals for testing. All mothers reported testing for HIV, syphilis, and hepatitis B on the recommendations of their midwives and doctors, and none reported testing on their own (Quotation 1/Q1).

Barriers at the policy level are related to testing services that are separate from ANC facilities. All mothers were tested at public health centers while performing antenatal check-ups at the midwives’ or doctors’ private practices (Q2).

### Community-level factors

Facilitators at the community level are involved in Non-Governmental (NGOs) and cross-sector involvement (Q3-Q4). Several NGOs played a crucial role in Bali. They have field outreach workers in hospitals and public health centers. Mothers infected with HIV mentioned having companions " from NGOs (Q3). This support is not only for mothers living with HIV but also for their partners, families, and newborns (Q4). This assistance is instrumental because it provides information on HIV infection, accompanies patients during treatment or examinations during pregnancy, and ensures regular medication intake (Q4).

Barriers at the community level included challenges for non-residents and fear of stigma and discrimination from the community (Q5-Q7). Non-residents of mothers who did not have any health insurance or could not afford medical care, childbirth, or other examinations could receive free health insurance from the local government, provided that they had a valid ID (KTP in Indonesia) in that area and a letter of inability to pay from the village head (Q6). On the other hand, mothers who are not native residents and do not have a valid ID with the same domicile face difficulties obtaining health insurance, where the payments are covered by the local government (known as the Healthy Indonesia Card/KIS) (Q5-Q6). They felt a significant financial burden when using health insurance that was paid independently every month (Q6). Some mothers expressed embarrassment about seeking care at public health centers or hospitals, particularly if the people around their community know who those seeking care are because of being infected with HIV and syphilis (Q7). However, mothers living with hepatitis B rarely express shame for seeking care at hospitals.

### Healthcare system or institutional-level factors

Facilitators in the healthcare system were related to tracking and follow-up by the assigned midwife in the area, good relationships between patients and healthcare providers, and satisfaction with hospital facilities and healthcare services (Q8-Q9). All pregnant mothers living with the virus mentioned receiving home visits from midwives once during pregnancy and after giving birth, both during pregnancy and postpartum, and visits to health facilities (Q8 and Q9). Mothers who did not undergo testing until the third trimester were visited by village midwives (Q8 and Q9). All pregnant mothers stated that they always followed and trusted all the information provided by the midwives and doctors conducting the examinations (Q10). Some mothers expressed comfort in giving birth at referral hospitals (public sector), even though they were far from their homes, owing to the quick response from healthcare professionals and air-conditioned rooms, even when using free health insurance (Q11).

Barriers to the healthcare system are related to long waiting times for hospital visits, insufficient information from healthcare providers regarding continuous care for mothers and infants, and administrative limitations in healthcare services for insurance users (Q12-Q15). Long waiting times, especially at the ticket counter, became inhibiting factors, as mothers had to spend the entire day at the hospital (Q12). Almost all participants mentioned that even if they arrived early, they still had to wait at the registration counter until noon, especially when seeking treatment and undergoing examinations at the hospital's healthcare services (Q12). However, almost all mothers expressed that during pregnancy checkups with midwives or specialist doctors, they did not receive clear information about the examinations or types of testing to be performed at public health centers, leading them to postpone testing because they were not provided with complete information regarding the types of testing and their purposes (Q13). Almost all mothers mentioned not receiving information that their child would be retested to determine whether the child was infected (Q14). The separation of ANC services from treatment services for mothers living with HIV and hepatitis B has prevented them from simultaneously accessing pregnancy examination services and taking medications. This was also influenced by private health insurance, which participants paid per month (*Badan Penyelenggara Jaminan Sosial Kesehatan*/BPJS), or public health insurance provided by the government (Kartu Indonesia Sehat/KIS) administration (Q15).

### Interpersonal-level factors

Facilitators at the interpersonal level included support, open communication, and free stigma from the partner or family (Q16-Q18). All mothers living with HIV, syphilis, and/or hepatitis B who had successfully undergone adequate treatment received support from their husbands and families (Q16). Husbands remind them to take medication and accompany them for prenatal checkups or treatment. (Q16). Families with financial and decision-making powers, such as fathers or siblings, play a crucial role in ensuring that mothers receive treatment according to the doctors' instructions. (Q17). Families also serve as substitutes for husbands in terms of collecting medication from public health centers for pregnant mothers or accompanying them for prenatal checkups and treatment when husbands cannot take leave at work or face other barriers. (Q17). Almost all pregnant mothers who had successfully received adequate treatment and had taken ARV medication until the time of the interview demonstrated openness regarding their condition to their sexual partners, husbands, and/or family members (Q18). Almost all mothers were open to family members who were more economically stable than economically unstable.

A significant barrier at the personal level, highlighted by the experiences of the participants, was the reluctance of sexual partners to undergo health screening (Q19). This fear of potential diagnoses can deter partners from participating in essential health checks, thereby not only compromising their own health but also impacting the effectiveness of programs aimed at eliminating mother-to-child transmission of infections such as HIV, syphilis, and hepatitis B.

### Individual-level factors

Facilitators at the individual level are related to mothers who desire a healthy baby and healthy lifestyle (Q20-Q21). Sufficient knowledge and self-acceptance were facilitators at the individual level (Q22-Q24). All mothers said that they wanted their children to be healthy and did not suffer from diseases like themselves. (Q20). All infected mothers had a desire to recover, so they always followed the advice of doctors and midwives, sought treatment regularly, and gave birth at the hospital according to the doctor's advice (Q21). All mothers received counseling from health providers and knew that the disease could be transmitted to the baby if they did not seek treatment (Q22 and Q23). However, not all mothers had good knowledge of the causes of maternal infection and comprehensive care for mother-baby-husband pairs (Q22 and Q23). Acceptance of the mother's illness affects her openness to her partner and/or family, and routine treatment (Q24).

Barriers at the individual level are related to a lack of administrative discipline. Some mothers did not have ID cards; therefore, they did not have KIS or national health coverage from the local government (Q25). The lack of an administrative order is a barrier for mothers to receive treatment immediately because it takes approximately one month to take care of the population administration and activate their KIS after receiving an ID card (Q25). Mothers who do not have ID cards or health insurance take approximately 4–10 weeks to start treatment for positive detection (Q25).

## Discussion

This study explored the barriers and facilitators of EMTCT. The findings of this study reveal that both Indonesia and Uganda face significant challenges in EMTCT, including healthcare system limitations and the need for effective community engagement [23]. However, Indonesia's struggle with bureaucratic barriers and financial constraints is in stark contrast to Uganda, where community mobilization and healthcare worker training have become more central issues [23]. Moreover, Nepal's approach to EMTCT, focusing on small-scale, community-focused interventions, offers a different perspective, emphasizing the importance of localized strategies [24]. These comparisons highlight the need for tailored approaches to EMTCT that consider each country's unique healthcare infrastructure, cultural dynamics, and public health literacy levels.

The Ministry of Health Regulation No. 57 of 2017 and Integrated ANC Guidelines in Indonesia are important for EMTCTs for HIV, syphilis, and hepatitis B [1, 9]. These regulations mandate early pregnancy screening as a crucial step toward managing infections in mothers, aligning with the WHO and MOH standards for universal testing in pregnant women [1, 4]. The success of EMTCT programs hinges on comprehensive support through human resources, finance, infrastructure, and supplies, as well as free screening and treatment policies, as demonstrated by countries such as Cuba and Thailand [16, 25]. However, challenges such as policy-induced testing barriers and the need for high screening coverage remain. At the community level, cross-sectoral involvement and community engagement facilitated the EMTCT. Community involvement can be achieved through mentorship programs or organizations created to promote treatment adherence [26, 27]. This is also supported by research results showing that peer group intervention by community volunteers increases HIV prevention knowledge [28]. However, challenges at The WHO community level, especially those faced by migrant mothers, include a greater likelihood of not receiving adequate treatment [5, 29, 30]. The WHO's efforts to validate EMTCT in each country, considering the stigma and human rights of mothers, still face obstacles, as revealed by research indicating that stigma, not only from partners, families, and society but also from healthcare providers and gender-related factors remain barriers to accessing preventive mother-to-child transmission services [31].

At the healthcare system or institutional level, tracking and follow-up by healthcare professionals, especially through home visits by midwives, trust in healthcare professionals, and satisfaction with healthcare services and hospitals, contribute to the success of EMTCT. The preparedness and adequacy of resources, facilities, and healthcare professionals' knowledge are critical factors in achieving EMTCT [8, 16, 32]. Inadequate counseling, insufficient information from healthcare providers, long waiting times, and stigma from healthcare professionals inhibit elimination [33].

At the interpersonal level, support from partners and families is a strong source of support, especially for treatment. This finding aligns with research findings that mothers with hepatitis B have strong knowledge and understanding of the disease and its transmission from mother to child. Motivated by ensuring their children’s health, they receive support from their partners and parents [20, 34]. Partner support in breaking the chain of transmission is crucial, especially for sexually transmitted infections [35, 36]. Both parties are expected to undergo treatment to prevent horizontal transmission. Despite partner support during pregnancy and childbirth, some mothers reported that their partners were unwilling to undergo testing, contrary to research indicating that male partners' involvement in HIV testing and counseling is influenced by their presence in antenatal care spaces with their wives [36].

At the individual level, the motivation to have a healthy child, recovery from illness, knowledge of preventing disease transmission from mother to child, and self-acceptance are facilitator factors in the success of EMTCT for HIV, syphilis, and hepatitis B. This finding is consistent with studies suggesting that positive perceptions about the benefits of testing for oneself and the baby influence testing and treatment continuity during pregnancy [16]. Individual experiences and the desire to maintain the baby's health contribute to mothers' adherence to treatment during pregnancy and breastfeeding [20]. A lack of comprehensive knowledge is an inhibiting factor in achieving EMTCT [37, 38]. Not having a national identity card complicates access to health insurance, leading to self-stigmatization and a lack of health insurance (costs), hindering access to EMTCT services. These challenges are consistent with various studies indicating that citizenship status, costs, stigma, and lack of information about sexual health are inhibiting factors in the screening and treatment of mothers living with any of the three infectious diseases [30].

The International Community of Women Living with HIV has submitted a report to the UN Working Group on Discrimination against Women, highlighting discrimination and abuse faced by women in healthcare [39]. Fear of mistreatment in maternal care deters women from seeking skilled care more significantly than factors such as cost or distance, especially in regions with high maternal mortality rates [39]. In this study, we found a different finding, where barriers to cost and distance to services related to bureaucratic issues were more vocally articulated by mothers than was fear of discrimination. Moreover, stigma, especially from health care providers, prevents mothers from accessing care. However, this study revealed that families and health workers provided support to mothers, and that mothers did not disclose social stigma. Hence, access to antenatal care in Bali Province shows that two out of three cities reached the coverage target (> 95%) [6]. Therefore, the problem landscape faced in EMTCT in Bali Province, Indonesia, is slightly different from the global problem, where bureaucracy is the main concern in accessing services compared to discrimination against women.

### Implications for practice

The implications of these studies include several key points: improving mothers’ knowledge remains crucial, even when adherence rates are satisfactory. Therefore, better and more continuous education programs are needed to provide in-depth information on prevention and treatment, which can strengthen public understanding, establish a solid foundation for long-term adherence, and reduce the stigma and discrimination against mothers. In this study, sufficient knowledge and self-acceptance were identified as facilitators of progress toward EMTCT. Besides receiving information from healthcare professionals, mothers also sought information about their condition online using their mobile phones. To ensure that mothers living with HIV, syphilis, and hepatitis B receive accurate and reliable information, it is essential to develop digital health literacy programs. Additionally, the involvement of NGOs and cross-sectoral collaboration (e.g., village heads and social services) played a crucial role in supporting these efforts. There is a strong correlation between digital health literacy and efforts to eradicate EMTCT. Digital health literacy strengthens community engagement in EMTCT efforts, facilitates the search for services, and improves treatment adherence, access, and comprehension of health information [40–42]. Multidisciplinary collaboration between multidisciplinary healthcare professionals and NGOs is essential for addressing the gap in limited sources. Moreover, further studies are needed to develop integrated care with technology based on mothers’ values and preferences in Bali, as a natural setting.

Considering the significant bureaucratic barriers to treatment access identified in our study, it is imperative to devise a comprehensive action plan to overcome these obstacles. This plan should prioritize the simplification of administrative processes to enhance the accessibility of EMTCT services. Key actions could include advocating policy reforms to reduce unnecessary red tape and facilitating collaboration between various governmental and nongovernmental entities to ensure a streamlined healthcare delivery system. Drawing inspiration from successful models in other Southeast Asian regions, including Thailand and Malaysia [10], these reforms must be supported by strong political commitment and leadership to foster a more efficient and responsive healthcare framework, ultimately improving outcomes for mother-to-child transmission prevention efforts. Moreover, the Healthy Indonesian Card for National Health Insurance Premium Assistance Recipients should be strengthened as an alternative option for individuals struggling to afford health insurance to reduce financial constraints and improve access to health care services.

Further studies with a cultural focus are necessary to understand why adherence rates remain high despite the potentially low levels of knowledge. By revealing the cultural and contextual elements that may elude detection through knowledge parameters in isolation, this study can serve as a foundation for developing more comprehensive and culturally appropriate strategies to address the requirements of the local population.

### Strengths and Limitations

Acknowledging the limitations in the accuracy of policies and healthcare practices from a patient's perspective is critical for ensuring patient-centered care. Patients often experience the impact of policies and healthcare decisions directly, and understanding their perspectives sheds light on the real-world effectiveness of these measures. The key limitation lies in the potential gap between policy intention and practical implementation. Patients’ perspectives also highlight the importance of their involvement in the decision-making process. Policies crafted without considering patient input may overlook crucial aspects of the patient experience, leading to potential inaccuracies in the relevance and effectiveness of the policy. Engaging patients in policymaking dialogue fosters a sense of ownership and ensures that policies align more closely with patients’ needs and preferences. However, acknowledging these limitations is crucial to maintaining transparency and managing patient expectations. Moreover, a patient's health literacy can influence the accuracy of healthcare information and the effectiveness of policies. Patients with varying levels of health literacy may interpret and apply healthcare information differently, affecting the outcomes of policy implementation. Financial insecurity is closely linked to lower health literacy, indicating that individuals struggling financially may benefit significantly from programs aimed at enhancing health literacy among people living with HIV [43]. Such programs can improve health outcomes by building confidence and resourcefulness, thus enabling individuals to better engage with health information. This approach not only educates, but also empowers, addressing both informational and financial barriers to effective health management [43].

Additionally, the study did not deeply explore the types, frequencies, or durations of support provided by NGOs and peer groups, thus limiting the understanding of effective support mechanisms. In addition, the findings are specific to the Indonesian context, and may not be generalizable to other regions with different cultural or health system dynamics. Therefore, future studies should conduct a detailed analysis of the different types of support provided by NGOs and peer groups to understand how variations affect the outcomes. Comparative studies across diverse geographical and cultural settings can enhance the generalizability of findings.

## Conclusion

This study identified key facilitators and barriers to healthcare access for mothers living with HIV, syphilis, and hepatitis B, emphasizing the importance of policy-driven screening, cross-sectoral support for free health insurance, positive healthcare relationships, partner involvement, and patient willingness for a healthy baby. Challenges include inadequate information from healthcare providers, complex health insurance processes, and self-stigmatization. This study suggests collaborative care interventions and integrated digital solutions to overcome these barriers, aiming to eliminate the mother-to-child transmission of these diseases by 2030.

### Supplementary Information

Supplementary Material 1.

## Data Availability

The data presented in this study are available in the manuscript.
