# Supplementary material for: Patient perspective on the elimination mother-to-child transmission of HIV, syphilis, and hepatitis B in Bali, Indonesia: a qualitative study
Source: BMC Public Health. 2024 Aug 20;24:2258. doi: 10.1186/s12889-024-19692-3 (PMC11337813; doi:10.1186/s12889-024-19692-3)
Supplement: Supplementary file 1 — Supplementary Material 1. [file 12889_2024_19692_MOESM1_ESM.docx]

**INTERVIEW GUIDELINES FOR MOTHERS LIVING WITH HIV**

1. **Preparation**

1. Contact the informant to confirm availability, time, and place for the interview.

2. Prepare supporting interview equipment, such as ensuring the recording device is functioning properly.

3. Dress respectfully and appropriately to honor the informant.

1. **Implementation**

1. Greet the respondent, and offer thanks for their willingness to be interviewed.

2. Introduce yourself.

3. Provide a brief explanation of the research background and objectives.

4. Explain that the interview results are solely for research purposes.

5. Clarify that all answers, comments, and information provided are important for the research and that there are no right or wrong answers.

6. Assure the respondent that all interview results and their identity will be kept confidential.

7. Request that the respondent provide honest and genuine responses and comments.

8. Thank the respondent at the end of the interview and ask for their willingness to be contacted if further information is needed.

1. **Respondent Information**

1. Day, date :

2. Time :

3. Place :

4. Respondent ID:

5. Gender :

6. Age :

7. Education :

8. Occupation :

9. Parity :

1. **Question Guide**

1. How is your health currently?

2. What health issues have you experienced so far?

3. When did you first find out you were living with HIV?

4. How did you feel when you first learned that you were living with HIV?

5. What have you done to manage your health since being diagnosed?

6. Do your partner or family know about your current health condition?

7. Have there been any changes in your relationship with your partner or family since your diagnosis was known?

8. How have your partner and family supported you since they became aware of your condition?

9. In what ways has their support been most meaningful to you?

10. Have you faced any obstacles while seeking pregnancy care or treatment?

11. How would you describe the quality of care you have received, especially in terms of facilities and healthcare services?

12. How is your communication with doctors, midwives, nurses, or other healthcare personnel? Do you feel anything is lacking?

13. How do you think people outside of your partner and family view individuals with HIV? Do you experience stigma, discrimination, or unpleasant treatment from them?

14. Do you receive support from any particular group or participate in educational programs? If yes, what do they provide?

15. How has their support impacted your health management?

16. Are you aware of any government policies designed to help patients like you?

17. Are there any challenges you face when accessing the care you need?

18. Do you think the current policies effectively support patients in your situation?

**INTERVIEW GUIDELINES FOR MOTHERS LIVING WITH SYPHILIS**

1. **Preparation**

1. Contact the informant to confirm availability, time, and place for the interview.

2. Prepare supporting interview equipment, such as ensuring the recording device is functioning properly.

3. Dress respectfully and appropriately to honor the informant.

1. **Implementation**

1. Greet the respondent, and offer thanks for their willingness to be interviewed.

2. Introduce yourself.

3. Provide a brief explanation of the research background and objectives.

4. Explain that the interview results are solely for research purposes.

5. Clarify that all answers, comments, and information provided are important for the research and that there are no right or wrong answers.

6. Assure the respondent that all interview results and their identity will be kept confidential.

7. Request that the respondent provide honest and genuine responses and comments.

8. Thank the respondent at the end of the interview and ask for their willingness to be contacted if further information is needed.

1. **Respondent Information**

1. Day, date :

2. Time :

3. Place :

4. Respondent ID:

5. Gender :

6. Age :

7. Education :

8. Occupation :

9. Parity :

1. **Question Guide**

1. How is your health currently?

2. What health issues have you experienced so far?

3. When did you first find out you were living with syphilis?

4. How did you feel when you first learned that you were living with syphilis?

5. What have you done to manage your health since being diagnosed?

6. Do your partner or family know about your current health condition?

7. Have there been any changes in your relationship with your partner or family since your diagnosis was known?

8. How have your partner and family supported you since they became aware of your condition?

9. In what ways has their support been most meaningful to you?

10. Have you faced any obstacles while seeking pregnancy care or treatment?

11. How would you describe the quality of care you have received, especially in terms of facilities and healthcare services?

12. How is your communication with doctors, midwives, nurses, or other healthcare personnel? Do you feel anything is lacking?

13. How do you think people outside of your partner and family view individuals with syphilis? Do you experience stigma, discrimination, or unpleasant treatment from them?

14. Do you receive support from any particular group or participate in educational programs? If yes, what do they provide?

15. How has their support impacted your health management?

16. Are you aware of any government policies designed to help patients like you?

17. Are there any challenges you face when accessing the care you need?

18. Do you think the current policies effectively support patients in your situation?

**INTERVIEW GUIDELINES FOR MOTHERS LIVING WITH HEPATITIS B**

1. **Preparation**

1. Contact the informant to confirm availability, time, and place for the interview.

2. Prepare supporting interview equipment, such as ensuring the recording device is functioning properly.

3. Dress respectfully and appropriately to honor the informant.

1. **Implementation**

1. Greet the respondent, and offer thanks for their willingness to be interviewed.

2. Introduce yourself.

3. Provide a brief explanation of the research background and objectives.

4. Explain that the interview results are solely for research purposes.

5. Clarify that all answers, comments, and information provided are important for the research and that there are no right or wrong answers.

6. Assure the respondent that all interview results and their identity will be kept confidential.

7. Request that the respondent provide honest and genuine responses and comments.

8. Thank the respondent at the end of the interview and ask for their willingness to be contacted if further information is needed.

1. **Respondent Information**

1. Day, date :

2. Time :

3. Place :

4. Respondent ID:

5. Gender :

6. Age :

7. Education :

8. Occupation :

9. Parity :

1. **Question Guide**

1. How is your health currently?

2. What health issues have you experienced so far?

3. When did you first find out you were living with hepatitis B?

4. How did you feel when you first learned that you were living with hepatitis B?

5. What have you done to manage your health since being diagnosed?

6. Do your partner or family know about your current health condition?

7. Have there been any changes in your relationship with your partner or family since your diagnosis was known?

8. How have your partner and family supported you since they became aware of your condition?

9. In what ways has their support been most meaningful to you?

10. Have you faced any obstacles while seeking pregnancy care or treatment?

11. How would you describe the quality of care you have received, especially in terms of facilities and healthcare services?

12. How is your communication with doctors, midwives, nurses, or other healthcare personnel? Do you feel anything is lacking?

13. How do you think people outside of your partner and family view individuals with hepatitis B? Do you experience stigma, discrimination, or unpleasant treatment from them?

14. Do you receive support from any particular group or participate in educational programs? If yes, what do they provide?

15. How has their support impacted your health management?

16. Are you aware of any government policies designed to help patients like you?

17. Are there any challenges you face when accessing the care you need?

18. Do you think the current policies effectively support patients in your situation?
